# Age-related differences in psychosocial risks among community mental health professionals

**DOI:** 10.3389/fpsyg.2026.1864668

**Published:** 2026-08-26

**Authors:** Coral Oliver, Irene Aliagas, Irene Mínguez-García, Inmaculada Mateo-Rodríguez, Eugenia González-de Molina, Elvira Pérez-Benítez

**Affiliations:** 1 Department of Social, Work and Differential Psychology, Faculty of Psychology, Universidad Complutense de Madrid, Madrid, Spain; 2 Escuela Andaluza de Salud Pública, Granada, Spain; 3 Departamento de Psicología social y de las organizaciones, UNED, Madrid, Spain; 4 Centro de Investigación Biomédica en Red de Epidemiología y Salud Pública (CIBERESP), Madrid, Spain; 5 IBS, Granada, Spain; 6 Fundación Pública Andaluza Para la Integración Social de Personas con enfermedad Mental (FAISEM), MP, Sevilla, Spain

**Keywords:** age, aging, job demands, job resources, mental health, occupational health, psychosocial risk, work

## Abstract

**Introduction:**

The progressive aging of the workforce poses important challenges for occupational health, particularly in emotionally demanding sectors such as community mental health services. Understanding age-related differences in psychosocial working conditions is crucial for promoting sustainable careers and successful aging at work.

**Objective and methods:**

This study examines psychosocial risk factors across five age groups (18–25, 26–35, 36–45, 46–55, ≥56) among community mental health professionals in Spain (*N* = 756). Psychosocial risks were assessed using a tailored instrument covering 13 factors, including: Leadership and communication, Interpersonal conflict at work, Work climate, Participation in decision-making, Organizational justice and recognition, Job stress, Work–family conflict, Role ambiguity, Work pace, Mental workload, Workplace violence, Job knowledge, and Work engagement. Age differences were analyzed using one-way ANOVAs with Bonferroni *post hoc* tests.

**Results:**

Overall, high levels of mental workload and work pace were observed across the sample. Significant differences were found in interpersonal conflict, job stress, work pace, and workplace violence. Employees aged 26–35 reported the most favorable psychosocial profile. Younger employees (18–25) reported the highest levels of interpersonal conflict. Older employees (46–55 and ≥56) experienced higher job stress. Work pace was highest among employees aged 46–55, and exposure to workplace violence was lower compared to younger and mid-career workers.

**Conclusion:**

These findings highlight age-related differences in psychosocial risks and underscore the need for age-sensitive organizational strategies in mental health care settings.

## 1 Introduction

The demographic aging of the workforce is a pressing concern in industrialized countries, driven by declining birth rates, rising life expectancy, and policies designed to delay retirement (Burr et al., 2017; Fattori et al., 2025; Shiri et al., 2025). As a result, the proportion of older employees is increasing, creating challenges such as labor shortages and imbalances between retirees and active workers (Ndjaboue et al., 2025). Workforce aging should not be understood solely as a demographic trend; functional aspects, including health, cognitive capacity, and work ability, also evolve over time and may show declines from midlife onwards (Fattori et al., 2025). Older workers also face additional vulnerabilities, including age-related stigma, stereotypes about productivity, and discrimination, which may accelerate premature exit from the labor market (Roscigno et al., 2022).

These challenges are particularly relevant in healthcare and social care sectors, which employ around 11% of the European Union workforce and is characterized by high emotional demands and frequent exposure to complex social interactions (Botey Gaudé et al., 2022; Jain, 2024). In these contexts, psychosocial risks, defined as aspects of work design, organization, and management that may harm employees’ psychological or physical wellbeing, are increasingly recognized as major determinants of occupational health. Psychosocial risks include aspects such as excessive workload, role conflict, lack of control, job insecurity, and exposure to workplace violence or harassment (Cañavate et al., 2023; Győri et al., 2024; Herz et al., 2026). From a European occupational safety and health perspective, psychosocial risks are recognized as work-related hazards that fall within employers’ responsibilities, although they remain insufficiently addressed in many organizational contexts, particularly in high-demand sectors such as health and social care (European Commission, 2023).

Exposure to psychosocial risks has been consistently linked to adverse outcomes, including depression, anxiety, burnout, and impaired cognitive and cardiovascular functioning (Fouquet et al., 2026). In healthcare settings, psychosocial risks also affect organizational performance, including patient safety, quality of care, and staff retention (García-Iglesias et al., 2021; Hall et al., 2016). Evidence from Spanish indicates a high prevalence of psychosocial risk exposure, particularly in relation to workload and psychological demands. These conditions have been associated with increased psychological distress, with nearly 41% of professionals at risk of mental health problems (García-Iglesias et al., 2021).

Among healthcare sectors, community mental health services are particularly affected due to their high emotional and social demands and frequent work with users in crisis situations (Győri et al., 2024; Pasquarella et al., 2022; Scanlan and Still, 2019). As employees age, their capacities, needs, and experiences evolve, influencing how they perceive and respond to workplace demands and resources (Burr et al., 2017; Cañavate et al., 2023). Accumulated work experience may provide older employees with internal resources to cope with job demands, increasing resilience under certain conditions (Cañavate et al., 2023). In addition, external resources such as autonomy and supervisory support are particularly important for older employees, helping to buffer stress associated with cognitive and physical demands (Pak et al., 2023; Shultz et al., 2010). Conversely, some psychological demands, such as high mental workload or time pressure, may be perceived as more threatening (Shultz et al., 2010).

The concept of sustainable work and healthy ageing at work emphasizes aligning job demands, resources, and individual capacities across the working life to maintain wellbeing and employability (Fattori et al., 2025; Pak et al., 2023). The Job Demands-Resources (JD-R) model provides a useful framework for understanding psychosocial risks, distinguishing between job demands that contribute to strain and job resources that support wellbeing and performance (Demerouti et al., 2001; Bakker and Demerouti, 2017). Within this model, job demands refer to aspects of work that require sustained effort and entail physiological or psychological costs, including workload, role ambiguity and time pressure (Lehmann and Bauer, 2025; Lesener et al., 2019; Pak et al., 2023). Job resources, in contrast, are aspects that help achieve work goals, reduce demands, and support personal growth, including autonomy, social support, and feedback (Herz et al., 2026; Pak et al., 2023). According to the JD-R model, excessive job demands can initiate a health-impairment pathway, leading to strain and burnout, whereas job resources support a motivational pathway that enhances engagement and performance (Lesener et al., 2019; Pasquarella et al., 2022). In this regard, identifying the specific factors that contribute to high demands, low support, or low control in particular occupational contexts, such as community mental health services, is essential for designing preventive measures that are appropriately tailored to these work environments.

Despite growing recognition of psychosocial risks in healthcare, most research has relied on aggregated samples, treating workers as a homogeneous group, and has paid limited attention to how these risks are distributed across the occupational lifespan or within specific contexts such as community mental health services (Broughton et al., 2025; Fouquet et al., 2026). This gap limits the identification of differentiated risk profiles and may hinder the development of targeted interventions tailored to employees’ career stages (Broughton et al., 2025). Importantly, psychosocial risks include excessive workload, role conflict, lack of control, job insecurity, and exposure to workplace violence or harassment (Győri et al., 2024; Herz et al., 2026). The perception and impact of these risks may vary with age; for example, older employees with temporary or precarious contracts may experience a pronounced resource loss cycle, increasing vulnerability to work-related stress (Ghezzi et al., 2020).

Given these considerations, the present study aimed to examine age-related differences in psychosocial risk factors among community mental health professionals in Spain, in order to identify which risks are more prevalent at different career stages and inform age-sensitive workplace interventions. Insights from this research may guide organizational strategies that support successful aging at work while safeguarding employee wellbeing and service quality.

## 2 Methods

### 2.1 Context

The study was conducted in a large, government-funded community mental health service in Andalucía, southern Spain. The service provides residential, day support, and community-based programs across multiple provinces, employing approximately 1,116 staff members across several service centers. The organization has a decentralized structure, with a presence in all eight provinces of the region and a central administrative body corresponding to the Regional Directorate, resulting in a large and territorially diverse organizational system. The study was initiated as a collaborative project between the university research team and the human resources and occupational health department. It was approved by the University Research Ethics Committee to which the authors are affiliated and conducted in accordance with the ethical principles of the Declaration of Helsinki (World Medical Association, 1964).

### 2.2 Sample

A total of 756 employees participated in the study, representing a response rate of approximately 71.12%, which strengthens the robustness and reliability of the dataset. The sociodemographic and occupational characteristics of the participants are presented in Table 1.

**Table 1:** Sociodemographic and occupational characteristics of the sample.

Variables	*N*	%
Gender		
Men	163	21.57
Women	577	76.32
Not reported	16	2.11
Age group		
18–25 years	12	1.59
26–35 years	52	6.88
36–45 years	208	27.51
46–55 years	336	44.44
≥56 years	148	19.58
Educational level		
Secondary education	10	1.32
High school	32	4.23
Professional education	335	44.31
Bachelor’s degree	199	26.32
Master’s degree or higher	180	23.81
Occupational category		
Service and maintenance staff	28	3.70
Occupational or sociocultural counselor	65	8.60
Residential support worker (with night shifts)	393	51.98
Residential support worker (without night shifts)	114	15.08
Administrative staff (supervisory role)	87	11.51
Administrative staff (non-supervisory role)	69	9.13
Department		
Administration	51	6.75
Employment program	36	4.76
Day support program	86	11.38
Residential program	583	77.12
Tenure in mental health services		
<1 year	123	16.27
1–5 years	139	18.39
5–10 years	104	13.76
10–20 years	219	28.97
>20 years	171	22.62

*N* = 756.

Most respondents were women (76.32%). Regarding age distribution, almost half of the sample was between 46 and 55 years old (44.44%), followed by those aged 36 to 45 years (27.51%). Participants reported diverse educational backgrounds, with a large proportion holding professional training qualifications (44.31%) or university degrees (50.13%). A substantial proportion of participants reported family care responsibilities: approximately 40% reported caring for dependent minors and nearly 19% reported caring for dependent adults, highlighting the potential relevance of work–life balance considerations in this workforce.

Regarding occupational roles, over half of the participants were residential support workers with night shifts (51.98%), followed by residential support workers without night shifts (15.08%) and administrative staff (20.64%). Most respondents worked within the residential care program (77.12%). Tenure in the current position indicates relatively stable employment trajectories, with more than half of the sample reporting over 10 years of experience (51.59%).

### 2.3 Measures and instruments

This study focused on examining age-related differences in psychosocial working conditions. Although socio-demographic and occupational information was collected to describe the sample, the primary variables of interest were participants’ age and their psychosocial risk profiles.

#### 2.3.1 Socio-demographic and occupational variables

Participants reported sex, educational level, professional category, department, and tenure in mental health services. To preserve anonymity, most items included a “prefer not to answer” option. These variables were used solely to characterize the sample and were not included in the main analyses.

Regarding age, participants were asked to indicate their age in one of these intervals: 18–25 years, 26–35 years, 36–45 years, 46–55 years, or more than 56 years. Age served as the independent variable in analyses examining differences across psychosocial factors.

#### 2.3.2 Psychosocial working conditions

Psychosocial factors at work were assessed using an instrument specifically developed for this study. The decision to develop a tailored measure was motivated by the need to capture psychosocial conditions particularly relevant to community mental health services, including exposure to workplace violence, relational dynamics, and organizational functioning across different service settings. The questionnaire was conceptually grounded in the Job Demands-Resources (JC-R) framework and drew on psychosocial dimensions commonly assessed in occupational health research.

The questionnaire comprised 65 items grouped into 13 factors, based on confirmatory factor analysis: Leadership and communication, Interpersonal conflict at work, Work climate, Participation in decision-making, Organizational justice and recognition, Job stress, Work–family conflict, Role ambiguity, Work pace, Mental workload, Workplace violence, Job knowledge, and Work engagement. The complete list of items, their allocation to each factor, and the standardized factor loadings are provided in Supplementary Table S1.

All items were rated on a 5-point Likert-type scale ranging from 1 (“strongly disagree”) to 5 (“strongly agree”). For example, items in the Interpersonal conflicts dimension included statements such as *“On occasion, a coworker or supervisor has behaved inappropriately toward me,”* whereas Job stress included items like *“Overall, I’d say my job is very stressful.”* Work pace was assessed with items such as “*The amount of work I have is usually consistent, and I can anticipate it in order to plan accordingly*,” and Workplace violence with statements like “*Assaults are a common occurrence in my daily life*.”

Scores for each psychosocial factor were computed as the mean of the corresponding items and subsequently rescaled to a 0–100 metric by multiplying by 20. Prior to computing the scale scores, all positively worded items were reverse-coded to ensure a consistent interpretation across all dimensions. Specifically, positively worded items were recoded so that higher values consistently reflected greater psychosocial risk, whereas negatively worded items retained their original coding. Consequently, higher scores on all factors indicate greater exposure to the psychosocial risk represented by that dimension. For example a high score on the Work climate factor reflects a poorer social and emotional environment within the team. Similarly, a high score on Job stress indicates elevated perceived stress, and a high score on Work Engagement reflects lower levels of engagement with one’s work.

The internal consistency of the factors was satisfactory, with Cronbach’s alpha coefficients ranging from 0.70 to 0.90. Construct validity was supported through both exploratory and confirmatory factor analyses. The exploratory factor analysis supported the expected dimensional structure, while the confirmatory factor analysis indicated a good model fit across all dimensions. Fit indices were consistently within recommended thresholds (Hu and Bentler, 1999; Kline, 2023), with Comparative Fit Index (CFI) values ranging from 0.975 to 0.999, Tucker–Lewis Index (TLI) from 0.950 to 0.999, Incremental Fit Index (IFI) from 0.975 to 0.999, Standardized Root Mean Square Residual (SRMR) from 0.010 to 0.076, and Goodness-of-Fit Index (GFI) from 0.991 to 0.999. These results provide strong support for the adequacy of the measurement model.

### 2.4 Procedure

All participants received detailed information about the objectives, procedures, and confidentiality of the studies, and provided informed consent for the use of their data. The anonymity of responses was guaranteed, and participation was voluntary. Employees were invited to complete an online survey. Data collection remained open for a period of 2 weeks, and the questionnaire required approximately 15 min to be completed.

### 2.5 Data analysis

Data were analyzed using IBM SPSS Statistics (version 28). First, descriptive statistics (means and standard deviations) were calculated for all study variables based on the rescaled scores, with particular attention to the distribution of age groups within the sample. Second, exploratory and confirmatory factor analyses were performed to examine the validity of the 13 psychosocial working conditions factors. Before conducting the group comparisons, the assumptions underlying one-way ANOVA were examined. Homogeneity of variances was assessed using Levene’s test and the assumption was met for all analyses based on the mean statistic (*p* > 0.05). Age was analyzed as a categorical variable to facilitate comparisons across meaningful career stages and to provide results that are readily interpretable from an occupational health perspective. Third, a series of one-way analyses of variance (ANOVAs) were conducted to assess differences in psychosocial factors across age groups. When significant effects were found, Bonferroni *post hoc* tests were applied to identify pairwise differences between groups. Effect sizes were estimated using eta squared (*η*^2^).

## 3 Results

### 3.1 Descriptive statistics

Descriptive analyses of the psychosocial factors indicated moderate to high mean scores across most dimensions (see Table 2). The highest scores were observed for mental workload (*M* = 71.29, *SD* = 14.21) and work pace (*M* = 60.66, *SD* = 15.94), suggesting elevated perceived demands in these areas. Job stress also showed relatively high levels (*M* = 57.06, *SD* = 21.07). In contrast, lower mean scores were found for workplace violence (*M* = 43.60, *SD* = 18.96), work climate (*M* = 47.00, *SD* = 18.13), and work–family conflict (*M* = 47.23, *SD* = 17.23). Intermediate values were observed for leadership and communication (*M* = 49.49, *SD* = 17.42), interpersonal conflict at work (*M* = 48.01, *SD* = 18.66), participation in decision-making (*M* = 51.74, *SD* = 16.99), organizational justice and recognition (*M* = 53.51, *SD* = 18.21), role ambiguity (*M* = 54.34, *SD* = 17.23), and job knowledge (*M* = 47.91, *SD* = 17.61). Work engagement showed comparatively lower mean levels (*M* = 36.55, *SD* = 11.79). Overall, the results suggest that job demands—particularly mental workload and work pace—are among the most prominent psychosocial factors in this sample.

**Table 2:** Descriptive statistics for each psychosocial factor.

Factors	*M*	*SD*
Leadership and communication	49.49	17.42
Interpersonal conflict at work	48.01	18.66
Work climate	47.00	18.13
Participation in decision-making	51.74	16.99
Organizational justice and recognition	53.51	18.21
Job stress	57.06	21.07
Work–family conflict	47.23	17.23
Role ambiguity	54.34	17.23
Work pace	60.66	15.94
Mental workload	71.29	14.21
Workplace violence	43.60	18.96
Job knowledge	47.91	17.61
Work engagement	36.55	11.79

*M* = Mean, *SD* = Standard Deviation.

**Figure 1:**
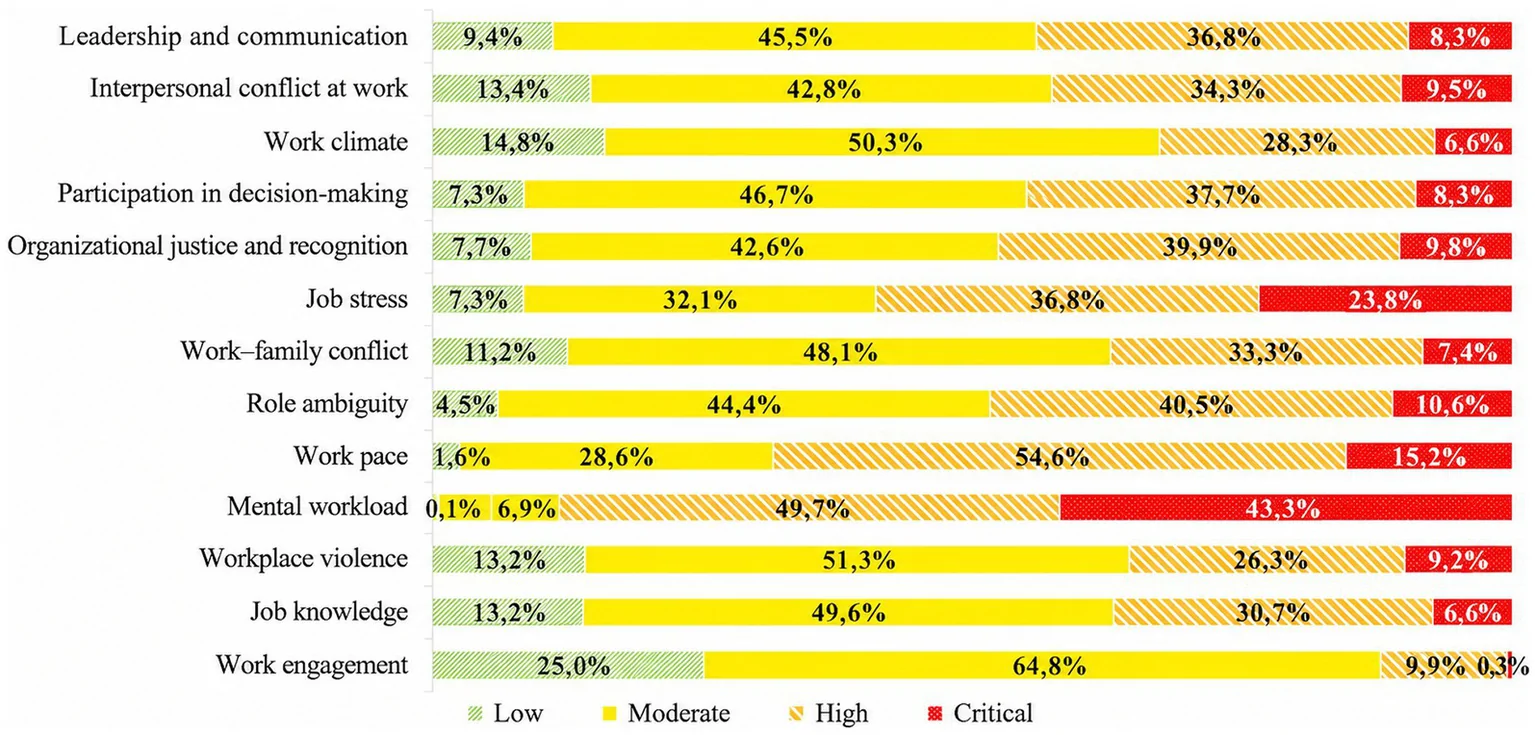
Distribution of psychosocial risk levels across participants. Created by the authors. Distributed under the terms of the Creative Commons Attribution License (CC BY) applied to this publication.

Figure 1 illustrates the distribution of psychosocial risk scores across the sample, organized into four exposure levels based on a rescaled 0–100 metric. Following a traffic-light color scheme, these levels correspond to increasing risk: low (0–25; green), moderate (26–50; yellow), high (51–75; orange), and critical (76–100; red). This approach allows for a clear visualization of the proportion of employees experiencing different degrees of psychosocial risk across the 13 assessed factors, facilitating the identification of areas where interventions may be most needed.

### 3.2 Mean differences

A series of one-way ANOVAs were conducted to examine age differences across the 13 psychosocial factors (see Table 3). Significant differences between age groups were found for interpersonal conflict at work, job stress, work pace, and workplace violence. According to Cohen (1992)‘s criteria, the observed effect sizes were small (*η*^2^ ranging from 0.016 to 0.026), indicating modest practical differences. The remaining psychosocial risks did not show statistically significant differences across age groups (all *p* > 0.05, *η*^2^ ≤ 0.012), indicating that these psychosocial risk are relatively stable across the occupational lifespan in this sector.

**Table 3:** Age differences in psychosocial risk factors.

Psychosocial factor	18–25 *M*(*SD*)	26–35 *M*(*SD*)	36–45 *M*(*SD*)	46–55 *M*(*SD*)	≥56 *M*(*SD*)	*F*	*p*	*η* ^2^
Leadership and communication	48.81 (15.04)	43.79 (17.7)	49.12 (16.95)	49.93 (16.86)	51.08 (19.13)	1.79	0.131	0.009
Interpersonal conflict at work	56.67 (25.23)	38.62 (16.23)	47.25 (18.63)	49.2 (18.77)	49 (17.77)	4.56	<0.001***	0.024
Work climate	52.5 (20.94)	43.65 (19.68)	46.88 (17.68)	47.81 (18.29)	46.08 (17.57)	0.99	0.414	0.005
Participation in decision-making	51.25 (17.47)	48.85 (17.62)	51.49 (16.89)	51.96 (16.54)	52.67 (17.97)	0.51	0.726	0.003
Organizational justice and recognition	52.08 (18.15)	48.17 (20.32)	53.75 (18.29)	53.97 (17.66)	54.12 (18.51)	1.24	0.293	0.007
Job stress	54.33 (25.72)	48.46 (20.62)	56.69 (20.37)	57.45 (20.72)	59.95 (22.01)	2.99	0.022*	0.016
Work–family conflict	48.67 (17.87)	41.46 (17.55)	47.58 (16.95)	47.44 (17.39)	48.19 (16.93)	1.63	0.168	0.009
Role ambiguity	52.92 (13.89)	50.87 (17.84)	54.62 (16.61)	54.30 (17.28)	55.41 (18.02)	0.70	0.592	0.004
Work pace	51.25 (15.69)	54.62 (15.59)	60.70 (15.11)	61.68 (15.77)	61.22 (17.05)	3.35	0.012*	0.018
Mental workload	65.33 (12.46)	66.46 (15.94)	71.90 (13.48)	71.79 (14.10)	71.49 (14.71)	2.25	0.063	0.012
Workplace violence	50.50 (17.19)	43.42 (17.32)	47.40 (19.25)	40.49 (18.35)	44.85 (19.55)	5.01	<0.001***	0.026
Job knowledge	51.25 (21.86)	51.44 (16.43)	47.04 (16.23)	49.02 (18.08)	45.14 (18.19)	2.02	0.092	0.011
Work engagement	35.83 (13.79)	34.90 (10.07)	36.85 (11.48)	36.65 (11.83)	36.55 (12.59)	0.30	0.884	0.002

Higher scores represent greater psychosocial risk after reverse coding of positively worded items. *M* = mean; *SD* = standard deviation. *p < 0.05, **p < 0.01, ***p < 0.001.

Bonferroni *post hoc* analyses were conducted to determine which age groups differed significantly for the psychosocial factors that showed significant ANOVA results.

For interpersonal conflict at work, ANOVA indicated significant differences among age groups, *F*(4, 751) = 4.56, *p* < 0.001, *η*^2^ = 0.024, 95% *CI* [0.004, 0.045]. *Post hoc* Bonferroni comparisons revealed that the youngest employees (18–25 years) reported significantly higher interpersonal conflict than employees aged 26–35 years (*MD* = 18.05, *SE* = 5.92, *p* = 0.024). Employees aged 26–35 years also reported lower conflict than those aged 36–45 years (*MD* = −8.63, *SE* = 2.87, *p* = 0.027), 46–55 years (*MD* = −10.59, *SE* = 2.76, *p* = 0.001), and ≥56 years (*MD* = −10.38, *SE* = 2.98, *p* = 0.005). Because the 18–25 age group included only 12 participants, findings involving this group should be interpreted with caution.

Significant age differences were also observed for job stress, *F*(4, 751) = 2.99, *p* = 0.022, *η*^2^ = 0.016, 95% *CI* [0.000, 0.033]. Post hoc analyses showed that employees aged 46–55 years reported higher stress than those aged 26–35 years (*MD* = 8.99, *SE* = 3.12, *p* = 0.041), and employees aged ≥56 years reported even higher stress compared to the 26–35 age group (*MD* = 11.48, *SE* = 3.38, *p* = 0.007).

For work pace, ANOVA indicated significant differences across age groups, *F*(4, 751) = 3.35, *p* = 0.012, *η*^2^ = 0.018, 95% *CI* [0.001, 0.036]. Post hoc Bonferroni comparisons revealed that employees aged 46–55 years reported higher perceived work pace than those aged 26–35 years (*MD* = 7.07, *SE* = 2.36, *p* = 0.028).

Finally, for workplace violence, ANOVA showed significant differences, *F*(4, 751) = 5.01, *p* < 0.001, *η*^2^ = 0.026, 95% *CI* [0.005, 0.048]. Post hoc comparisons indicated that employees aged 36–45 years reported higher exposure than those aged 46–55 years (*MD* = 6.91, *SE* = 1.66, *p* < 0.001).

## 4 Discussion

This study examined age-related differences in psychosocial risk factors among community mental health professionals in Spain. Descriptive analyses indicated moderate to high exposure across most psychosocial dimensions, particularly mental workload, work pace, and job stress. This suggests that cognitive and temporal demands are especially salient in this sector, consistent with previous findings in community mental health (Pasquarella et al., 2022; Scanlan and Still, 2019) and the broader Spanish healthcare context (Cañavate et al., 2023; García-Iglesias et al., 2021; Narbona-Gálvez et al., 2025). Age differences were observed in interpersonal conflict, job stress, work pace, and workplace violence, highlighting that the type of psychosocial risk varies across career stages.

### 4.1 Age differences in psychosocial risks

The analysis by age groups revealed significant differences in several psychosocial risk factors, indicating that age is associated with differences in the type of psychosocial risks experienced.

The youngest employees (18–25 years) reported the highest levels of interpersonal conflict at work. This result is consistent with evidence suggesting that early-career professionals face challenges in navigating workplace relationships and establishing authority within organizational structures (Győri et al., 2024; Narbona-Gálvez et al., 2025; Salmela-Aro and Upadyaya, 2018). However, this finding should be interpreted with caution because the youngest age group was represented by only 12 participants. Consequently, although the observed pattern is theoretically plausible and consistent with previous literature, it requires confirmation in studies including a larger number of younger workers. Employees aged 26–35 reported the lowest levels of interpersonal conflict, which may reflect a combination of increased experience, better adaptation to organizational norms, and more stable integration within teams (Ghezzi et al., 2020; Győri et al., 2024). These results suggest that relational challenges may be more pronounced at younger ages, when adaptation processes are still ongoing and team stability may be lower.

Regarding workplace violence, significant differences were observed between employees aged 36–45 and 46–55, with the 36–45 group reporting higher exposure. Notably, the youngest employees (18–25) showed comparatively lower scores, although these differences were not statistically significant in *post hoc* analyses. This pattern suggests that exposure to workplace violence may be more closely linked to the type and intensity of job demands than to age per se. In community mental health services, professionals often work with users in crisis and under high time constraints, conditions than increase the likelihood of encountering aggressive behaviors (Győri et al., 2024; Pasquarella et al., 2022). In this context, employees in certain age groups may be more frequently involved in complex, frontline, or high-pressure roles, which could increase their exposure to aggressive behaviors from service users.

Job stress and perceived work pace increased progressively with age. Employees aged 46–55 and ≥56 reported higher levels than younger colleagues, consistent with the accumulation of responsibilities, including administrative tasks, mentoring of junior staff, and complex case management tasks (Dūdiņa and Martinsone, 2025; Pak et al., 2023; Shultz et al., 2010). These findings indicate that older employees may face higher cognitive, emotional, and temporal demands. The observed pattern is consistent with the idea that the distribution of psychosocial risks may differ across age groups, with relational challenges being more frequently reported by younger ages and workload intensity and sustained stress by older ages (Győri et al., 2024; Salmela-Aro and Upadyaya, 2018).

Taken together, these results suggest the possibility of two differentiated psychosocial risk profiles across the occupational lifespan: a “youth profile,” characterized by interpersonal conflict and relational challenges, and a “mid- to late-age profile,” defined by increased stress, and sustained task demands. This differentiation highlights the moderating role of age in shaping the type of psychosocial risk to which employees are exposed, emphasizing the importance of age-sensitive strategies to support wellbeing and performance across the working life (Cañavate et al., 2023; Fattori et al., 2025). Although statistically significant, the observed effects were generally small according to Cohen’s criteria, suggesting modest differences between age groups. Consequently, these findings should be interpreted as general trends rather than sharply differentiated psychosocial profiles and should not constitute the sole basis for organizational decision-making.

### 4.2 Theoretical implications

The observed pattern reflects the association between age and occupational demands, suggesting that psychosocial risks may not be uniformly distributed across age groups (Salmela-Aro and Upadyaya, 2018). Specifically, relational demands such as interpersonal conflict appear to be more salient among younger employees, whereas demands related to work pace and job stress become more prominent at older ages, likely due to the accumulation of responsibilities over time.

These findings are consistent with the Job Demands-Resources model (Demerouti et al., 2001), which emphasize the importance of aligning organizational resources with job demands to maintain wellbeing and productivity across the working life (Cañavate et al., 2023; Lesener et al., 2019; Pasquarella et al., 2022). They also suggest that the relative salience of specific demands may vary as a function of age. This perspective aligns with lifespan and successful aging at work frameworks, which emphasize the need to adapt work conditions to individuals’ changing capacities, motives, and resources over time (Salmela-Aro and Upadyaya, 2018). These findings are consistent with the literature on successful aging at work, which emphasizes that employees’ needs, resources, and occupational experiences evolve across career stages while highlighting the importance of avoiding age-based stereotypes and discriminatory assumptions when designing organizational policies (Roscigno et al., 2022; Shultz et al., 2010).

Furthermore, the absence of significant age differences in factors such as leadership and communication, work climate, and work engagement suggests that the experience or perception of these aspects may be relatively stable across the occupational lifespan. This stability may reflect organizational or contextual factors that act as shared resources, buffering age-related differences in other psychosocial risks (Fattori et al., 2025; Narbona-Gálvez et al., 2025).

### 4.3 Practical recommendations

From a practical standpoint, the findings support the need for age-sensitive approaches to psychosocial risk management in community mental health services. Rather than adopting uniform interventions, organizations may benefit from tailoring strategies to the specific psychosocial challenges associated with different age groups (Cañavate et al., 2023; Ndjaboue et al., 2025; Pak et al., 2023).

For employees under 25 years, who show higher levels of interpersonal conflict, interventions may focus on strengthening induction processes, mentorship, and training in interpersonal communication and conflict management. Preventive measures addressing workplace violence and clear reporting procedures may also be particularly relevant in this group.

For employees over 36, who report higher levels of stress, work pace, and mental workload, organizational strategies aimed at task distribution, and stress management may be especially beneficial. Measures such as adequate staffing, structured work organization, and scheduled breaks may help reduce sustained demands.

In addition, cross-cutting interventions—such as flexible work arrangements, work–life balance initiatives, and supportive leadership—may contribute to maintaining wellbeing across all age groups, particularly in emotionally demanding care settings (Wang et al., 2022; Scanlan and Still, 2019). Importantly, age-sensitive interventions should be implemented in ways that address employees’ needs without reinforcing age-based stereotypes or discriminatory practices (Roscigno et al., 2022).

### 4.4 Limitations and future research

Despite a relatively large sample and high response rate, several limitations should be acknowledged. First, the cross-sectional design restricts causal inferences; therefore, the observed differences represent associations between age groups at a single point in time and should not be interpreted as developmental trajectories or evidence that psychosocial risks change as employees age. Longitudinal studies are needed to track psychosocial risks across the occupational lifespan (Ghezzi et al., 2020; Lesener et al., 2019). Second, the study was conducted in a single regional context (Andalusia, Spain), which may limit generalizability of the findings to other organizational or cultural settings. Consequently, the results should be interpreted as reflecting the characteristics of one specific organizational context rather than being broadly representative of community mental health services. Caution is therefore warranted when generalizing these findings to other mental health settings, healthcare systems, or countries with different organizational structures, workforce characteristics, and psychosocial regulations. Third, although some age-related differences were statistically significant, the effect sizes were small (*η*^2^ = 0.016–0.026), indicating that these differences, while meaningful, are modest in practical terms. This underscores the need for cautious interpretation regarding the magnitude of age effects on psychosocial risk factors. Additionally, the use of a study-specific instrument, although theoretically grounded in the JD-R framework and supported by satisfactory psychometric properties, may limit comparability with studies using standardized measures. Finally, the analyses focused on comparisons between age groups and did not control for other occupational or demographic characteristics. Although variables such as tenure, occupational category, gender, supervisory responsibilities, and type of work may also influence psychosocial risk exposure, they were not included as covariates because the primary aim of the study was to provide an exploratory description of age-related differences. Moreover, most of these variables were categorical and highly associated with age, resulting in numerous small subgroups that limited the feasibility and interpretability of adjusted analyses. Consequently, the observed age-related patterns should not be interpreted as attributable solely to chronological age, but may partly reflect differences in role configurations and exposure profiles within the organization.

Future research should adopt longitudinal designs to examine the dynamic interplay between age, tenure, and psychosocial working conditions over time, as current evidence highlights the need to better understand how these factors evolve across the working life (Ndjaboue et al., 2025). Future studies should also employ multivariable analytical approaches, such as regression models or ANCOVA where appropriate, to better disentangle the contribution of chronological age from that of tenure, occupational category, supervisory responsibilities, and other job characteristics. The present study focused exclusively on psychosocial working conditions and did not assess employee outcomes. Further studies should also explore whether age-related differences in psychosocial working conditions are associated with outcomes such as burnout, job satisfaction, and retention, given the well-established links between working conditions, employee wellbeing and organizational outcomes (Dūdiņa and Martinsone, 2025; Fattori et al., 2025). Moreover, it would be interesting to examine whether the observed associations between age and psychosocial risk factors are influenced by other variables, such as job tasks, role responsibilities, or degree of contact with service users. For example, tasks involving more direct interaction with patients may increase exposure to workplace violence, raising the question of whether certain age groups are more frequently involved in these roles. Addressing these factors would help clarify the mechanisms underlying age-related differences in psychosocial risk exposure.

## 5 Conclusion

This study provides evidence that psychosocial working conditions are associated with age across the occupational lifespan in community mental health services. Younger employees experience higher interpersonal conflict and, in mid-career, greater exposure to workplace violence, whereas older employees face higher stress, work pace, and cognitive-emotional demands. These findings highlight the relevance of age-sensitive organizational approaches to support employee wellbeing and promote sustainable careers in emotionally demanding care settings. Tailoring strategies to the psychosocial challenges characteristic of different age groups can foster sustainable employment and enhance both staff and service-user outcomes in emotionally demanding care professions. Moreover, this study provides preliminary evidence to inform the design of differentiated coping and prevention strategies according to age, offering useful guidance for organizational prevention services in assessing and prioritizing interventions adapted to workers’ specific needs.

## Data Availability

The data that support the findings of this study are not publicly available due to confidentiality, but can be obtained from the corresponding author upon reasonable request.
