# Supplementary material for: Age-related differences in psychosocial risks among community mental health professionals
Source: Front Psychol. 2026 Aug 26;17:1864668. doi: 10.3389/fpsyg.2026.1864668 (PMC13553237; doi:10.3389/fpsyg.2026.1864668)
Supplement: Supplementary file 1 [file Table_1.docx]

**Supplementary Table S1.** *Results From a Confirmatory Factor Analysis of the Psychosocial Working Conditions Questionnaire.*

| Items and Dimensions | Factor loading | | | | | | | | | | | | |
| --- | --- | --- | --- | --- | --- | --- | --- | --- | --- | --- | --- | --- | --- |
|  | 1 | 2 | 3 | 4 | 5 | 6 | 7 | 8 | 9 | 10 | 11 | 12 | 13 |
| Factor 1: Leadership and communication |  |  |  |  |  |  |  |  |  |  |  |  |  |
| 47. I feel supported by my supervisors in complex situations. (R) | .748 |  |  |  |  |  |  |  |  |  |  |  |  |
| 64. Communication between management and the rest of the staff is clear and open. (R) | .717 |  |  |  |  |  |  |  |  |  |  |  |  |
| 11. What FAISEM says is consistent with what it actually does in daily practice. (R) | .669 |  |  |  |  |  |  |  |  |  |  |  |  |
| 2. My supervisors have supported me after incidents of violence. (R) | .658 |  |  |  |  |  |  |  |  |  |  |  |  |
| 10. I receive support and assistance when I need it. (R) | .638 |  |  |  |  |  |  |  |  |  |  |  |  |
| 12. Internal policies (leave, work-life balance, etc.) are applied using clear, fair criteria and in a way that is equitable for everyone. (R) | .627 |  |  |  |  |  |  |  |  |  |  |  |  |
| 33. The information FAISEM provides me regarding training and professional development is adequate. (R) | .607 |  |  |  |  |  |  |  |  |  |  |  |  |
| Factor 2: Interpersonal conflict at work |  |  |  |  |  |  |  |  |  |  |  |  |  |
| 25. A coworker or supervisor has made comments about me that made me feel bad. |  | -.769 |  |  |  |  |  |  |  |  |  |  |  |
| 8. I have experienced or witnessed physical or psychological violence or harassment in my workplace. |  | -.752 |  |  |  |  |  |  |  |  |  |  |  |
| 38. On occasion, a coworker or supervisor has behaved inappropriately toward me. |  | -.734 |  |  |  |  |  |  |  |  |  |  |  |
| 4. At FAISEM, interpersonal conflicts and discrimination occur. |  | -.592 |  |  |  |  |  |  |  |  |  |  |  |
| 29. Taking initiative can backfire on me. |  | -.592 |  |  |  |  |  |  |  |  |  |  |  |
| Factor 3: Work climate |  |  |  |  |  |  |  |  |  |  |  |  |  |
| 52. The atmosphere on my team is collaborative. (R) |  |  | .757 |  |  |  |  |  |  |  |  |  |  |
| 60. Communication within the team is clear and open. (R) |  |  | .721 |  |  |  |  |  |  |  |  |  |  |
| 39. I feel like I’m part of a team where we support one another. (R) |  |  | .687 |  |  |  |  |  |  |  |  |  |  |
| 13. There’s a good atmosphere with my coworkers and supervisors. (R) |  |  | .474 |  |  |  |  |  |  |  |  |  |  |
| Factor 4: Participation in decision-making |  |  |  |  |  |  |  |  |  |  |  |  |  |
| 17. There are times—such as meetings, assemblies, breaks, etc.—when we can talk about how we feel or share our experiences. (R) |  |  |  | .672 |  |  |  |  |  |  |  |  |  |
| 16. I can take the initiative, and my suggestions are considered. (R) |  |  |  | .665 |  |  |  |  |  |  |  |  |  |
| 36. I can express my ideas respectfully in my work environment. (R) |  |  |  | .554 |  |  |  |  |  |  |  |  |  |
| 51. My work is recognized and appreciated by others. (R) |  |  |  | .530 |  |  |  |  |  |  |  |  |  |
| Factor 5: Organizational justice and recognition |  |  |  |  |  |  |  |  |  |  |  |  |  |
| 21. I feel like I put in a lot of effort, but my work isn’t sufficiently recognized or valued. |  |  |  |  | -.660 |  |  |  |  |  |  |  |  |
| 44. The distribution of tasks among the team isn’t balanced. |  |  |  |  | -.617 |  |  |  |  |  |  |  |  |
| 46. At work, I sometimes receive conflicting instructions. |  |  |  |  | -.549 |  |  |  |  |  |  |  |  |
| 3. I am treated fairly and receive appropriate recognition for my achievements. (R) |  |  |  |  | .525 |  |  |  |  |  |  |  |  |
| Factor 6: Job stress |  |  |  |  |  |  |  |  |  |  |  |  |  |
| 26. My job affects my emotional and physical health. |  |  |  |  |  | .793 |  |  |  |  |  |  |  |
| 45. I feel completely exhausted and burned out from my job. |  |  |  |  |  | .760 |  |  |  |  |  |  |  |
| 49. Overall, I would say my job causes me a lot of stress. |  |  |  |  |  | .754 |  |  |  |  |  |  |  |
| 57. I often feel nervous, tired, or physically and emotionally drained. |  |  |  |  |  | .721 |  |  |  |  |  |  |  |
| 24. My job is emotionally intense and affects my personal well-being. |  |  |  |  |  | .674 |  |  |  |  |  |  |  |
| Factor 7: Work–family conflict |  |  |  |  |  |  |  |  |  |  |  |  |  |
| 19. I have a hard time disconnecting from work when my workday ends. |  |  |  |  |  |  | .803 |  |  |  |  |  |  |
| 61. I usually take work home with me. |  |  |  |  |  |  | .785 |  |  |  |  |  |  |
| 20. I can’t seem to separate my work time from my personal time. |  |  |  |  |  |  | .762 |  |  |  |  |  |  |
| 42. I have a hard time putting my work problems out of my mind. |  |  |  |  |  |  | .675 |  |  |  |  |  |  |
| 50. I usually have to deal with personal matters while I’m working or have to do work-related tasks outside of my regular hours. |  |  |  |  |  |  | .617 |  |  |  |  |  |  |
| Factor 8: Role ambiguity |  |  |  |  |  |  |  |  |  |  |  |  |  |
| 27. In my position, there is no clear organization of daily tasks. |  |  |  |  |  |  |  | .665 |  |  |  |  |  |
| 9. I am forced to constantly improvise to keep everything running smoothly at work. |  |  |  |  |  |  |  | .664 |  |  |  |  |  |
| 48. I start each shift without a clear idea of what I’m going to encounter. |  |  |  |  |  |  |  | .634 |  |  |  |  |  |
| 40. I find it difficult to stay focused because of the volume and variety of my tasks. |  |  |  |  |  |  |  | .583 |  |  |  |  |  |
| Factor 9: Work pace |  |  |  |  |  |  |  |  |  |  |  |  |  |
| 41. My workload is usually consistent, and I can anticipate it so I can plan ahead. (R) |  |  |  |  |  |  |  |  | -.681 |  |  |  |  |
| 65. I find it difficult to finish my tasks on time. |  |  |  |  |  |  |  |  | .645 |  |  |  |  |
| 63. I work at a pace I can handle without feeling overwhelmed. (R) |  |  |  |  |  |  |  |  | -.541 |  |  |  |  |
| 1. I have enough time to complete my daily tasks. (R) |  |  |  |  |  |  |  |  | -.523 |  |  |  |  |
| Factor 10: Mental workload |  |  |  |  |  |  |  |  |  |  |  |  |  |
| 37. To do my job well, I have to handle complex information and a great deal of knowledge. |  |  |  |  |  |  |  |  |  | .783 |  |  |  |
| 31. I perform tasks that go beyond what is expected of my job category. |  |  |  |  |  |  |  |  |  | .556 |  |  |  |
| 30. My job requires me to make difficult decisions frequently. |  |  |  |  |  |  |  |  |  | .549 |  |  |  |
| 54. In general, the work I do is easy. (R) |  |  |  |  |  |  |  |  |  | -.515 |  |  |  |
| 7. I have to handle several tasks at the same time. |  |  |  |  |  |  |  |  |  | .504 |  |  |  |
| Factor 11: Workplace violence |  |  |  |  |  |  |  |  |  |  |  |  |  |
| 55. Service users have sometimes grabbed or touched me in an aggressive or inappropriate manner. |  |  |  |  |  |  |  |  |  |  | .805 |  |  |
| 35. Service users have sometimes yelled at me, pushed me, shoved me, or spat at me. |  |  |  |  |  |  |  |  |  |  | .774 |  |  |
| 22. Service users rudely interrupt my work. |  |  |  |  |  |  |  |  |  |  | .756 |  |  |
| 59. I have felt unsafe at my job for fear of being subjected to aggressive behavior. |  |  |  |  |  |  |  |  |  |  | .744 |  |  |
| 28. Service users express their anger by raising their voices or complaining. |  |  |  |  |  |  |  |  |  |  | .681 |  |  |
| 32. Aggressive behavior is common in my day-to-day work. |  |  |  |  |  |  |  |  |  |  | .674 |  |  |
| 18. Service users show their anger by destroying doors, windows, walls, etc. |  |  |  |  |  |  |  |  |  |  | .666 |  |  |
| 34. Service users get angry with me because of delays in care. |  |  |  |  |  |  |  |  |  |  | .636 |  |  |
| 14. Service users often make jokes or in poor taste comments to me. |  |  |  |  |  |  |  |  |  |  | .615 |  |  |
| 53. Service users unjustifiably accuse me of failures or complications. |  |  |  |  |  |  |  |  |  |  | .589 |  |  |
| Factor 12: Job knowledge |  |  |  |  |  |  |  |  |  |  |  |  |  |
| 5. I have received specific training on handling crisis situations, disruptive events, or workplace harassment. (R) |  |  |  |  |  |  |  |  |  |  |  | .882 |  |
| 43. I am familiar with the protocols for how to respond in crisis situations, cases of harassment, etc. (R) |  |  |  |  |  |  |  |  |  |  |  | .874 |  |
| 23. If I were to be the victim of an assault, I know how to respond and who to turn to. (R) |  |  |  |  |  |  |  |  |  |  |  | .853 |  |
| 15. The training I received on managing crisis situations at work has been very useful to me. (R) |  |  |  |  |  |  |  |  |  |  |  | .808 |  |
| Factor 13: Work engagement |  |  |  |  |  |  |  |  |  |  |  |  |  |
| 62. My level of satisfaction with my job, taking all aspects into account, is very high. (R) |  |  |  |  |  |  |  |  |  |  |  |  | .705 |
| 6. I am proud of my work and believe it adds value to the company. (R) |  |  |  |  |  |  |  |  |  |  |  |  | .755 |
| 58. I feel committed to my profession. (R) |  |  |  |  |  |  |  |  |  |  |  |  | .749 |
| 56. I believe my tasks are important and meaningful. (R) |  |  |  |  |  |  |  |  |  |  |  |  | .711 |

*Note.* *N* = 756. Reverse-scored items are denoted with an (R).
